# Expanding the horizon of continuous glucose monitoring into the future of pediatric medicine

**DOI:** 10.1038/s41390-024-03573-x

**Published:** 2024-09-21

**Authors:** Lourdes Morales-Dopico, Sarah A. MacLeish

**Affiliations:** 1 Pediatric Endocrinology Fellow, CWRU School of Medicine, University Hospitals Cleveland Medical Center, Rainbow Babies and Children’s Hospital, Cleveland, OH USA; 2 Associate Professor of Pediatrics, Pediatric Endocrinology, CWRU School of Medicine, University Hospitals Cleveland Medical Center, Rainbow Babies and Children’s Hospital, Cleveland, OH USA

## Abstract

**Abstract:**

Glucose monitoring has rapidly evolved with the development of minimally invasive continuous glucose monitoring (CGM) using interstitial fluid. It is recommended as standard of care in the ambulatory setting, nearly replacing capillary glucose testing in those with access to CGM. The newest CGM devices continue to be smaller and more accurate, and integration with automated insulin delivery systems has further revolutionized the management of diabetes, leading to successful improvements in care and quality of life. Many studies confirm accuracy and application of CGM in various adult inpatient settings. Studies in adult patients increased during the COVID 19 Pandemic, but despite reassuring results, inpatient CGM use is not yet approved by the FDA. There is a lack of studies in inpatient pediatric settings, although data from the NICU and PICU have started to emerge. Given the exponential increase in the use of CGM, it is imperative that hospitals develop protocols for CGM use, with a need for ongoing implementation research. In this review we describe how CGM systems work, discuss benefits and barriers, summarize research in inpatient pediatric CGM use, explore gaps in research design along with emerging recommendations for inpatient use, and discuss overall CGM utility beyond outpatient diabetes management.

**Impact:**

- Current CGM systems allow for uninterrupted monitoring of interstitial glucose excursions, and have triggered multiple innovations including automated insulin delivery.
- CGM technology has become part of standard of care for outpatient diabetes management, endorsed by many international medical societies, now with significant uptake, replacing capillary glucose testing for daily management in patients with access to CGM technology.
- Although CGM is not approved by the FDA for inpatient hospital use, studies in adult settings support its use in hospitals. More studies are needed for pediatrics.
- Implementation research is paramount to expand the role of CGM in the inpatient setting and beyond.

## Introduction

The introduction of continuous glucose monitoring (CGM) technology, utilizing interstitial fluid as a new compartment for measuring glucose, has evolved rapidly over the last two decades. Outpatient diabetes management for both children and adults has been transformed and optimized by CGM, and more recently this technology is also leading to advancements and changes in inpatient glucose monitoring and management. In the early 2000s research around diabetes technology, namely the CGM, was geared towards its validity, utilization and integration into daily long-term management of diabetes. The integration of CGM coupled with intense multiple daily insulin injections or continuous subcutaneous insulin infusion (insulin pump) is rapidly becoming part of the standard of care in the ambulatory setting for glycemic control, as recommended by the International Society of Pediatric and Adolescent Diabetes (ISPAD), the American Diabetes Association (ADA) and the European Association for the Study of Diabetes (EASD).^1–3^

**Fig. 1:**
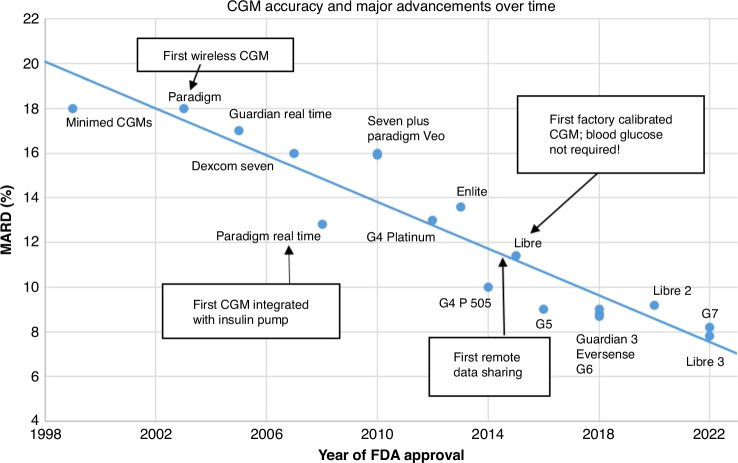
CGM accuracy and major advancements over time. Modified with permission from Bailey et al. The lower the MARD the better the accuracy.

The pace of advancements in CGM technology has been moving swiftly (Fig. 1), starting with the first FDA approved device, the CGMS System Gold by Medtronic Minimed in 1999.^4^ This was a blinded CGM that could be worn for 3 days with retrospective data collection via device download by the physician. A few years later in 2004 Medtronic introduced the Guardian real-time CGM into the market, which was capable of providing hypoglycemia or hyperglycemia alerts. The appearance of Dexcom and Freestyle Libre CGMs followed in 2006 and 2008, respectively. All of the initial CGM devices required calibration with a fingerstick glucose to improve accuracy, and treatment decisions could not be made without a confirmatory blood glucose. Over time, the accuracy of all of these sensors improved. By 2016 Abbott introduced the FreeStyle Libre Pro, the first factory-calibrated CGM,^5^ but still blinded. That same year, the FDA approved Dexcom G5 to be used as a non-adjunctive tool for management of diabetes, which meant a fingerstick glucose was not needed for treatment decisions,^6^ although it still required calibration. The following year, Freestyle Libre flash glucose monitoring was the first factory-calibrated device also approved for non-adjunctive use. Another novel technology was the first fully implantable glucose sensor, Eversense, which was FDA approved in 2018 for adults only. It requires a minor procedure to insert the sensor, and a transmitter is still worn on the body. Originally approved for 3 month use, it is now approved for 6 month use.

**Fig. 2:**
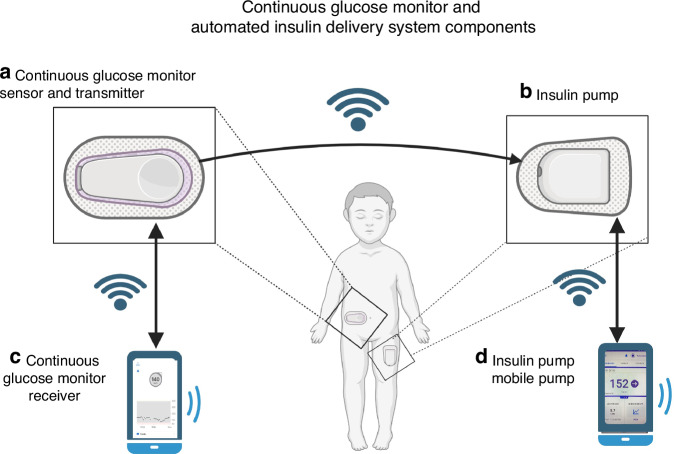
Continuous glucose monitor and automated insulin delivery system components. **a** Continuous glucose monitors include the sensor, which is a very thin filament that is inserted in the subcutaneous tissue and measures interstitial glucose, as well as a transmitter that stores and sends the glucose data to be displayed. The newest generations of CGMs have a disposable sensor and transmitter that are combined in one unit. **b** The CGM signal travels via bluetooth to the insulin pump. The automated insulin delivery algorithm uses the CGM data to predict future glucose and calculate insulin needs to try to keep glucose in the target range. The insulin pump continuously delivers rapid acting insulin based on the algorithms calculations. **c** CGM data is sent via Bluetooth to the receiver. The receiver can be a separate unit that is designed to display the CGM data, or can be a mobile app installed on a smartphone. If the receiver is a on a smartphone, the phone can then send the signal and display the same data to “followers”, such as parents or partners of the person living with diabetes. This feature is commonly used by parents when children are in school or with other caregivers. **d** The CGM and insulin data for the automated insulin delivery system may be displayed on the pump screen, or depending on the system, may be displayed on a mobile phone app. Often, patients use one mobile phone for both the CGM app and the insulin pump app.

These advances were a breakthrough in diabetes technology, increasing the use of CGM^7^ and leading the way for the development of automated systems for insulin delivery. Since then there have been newer generations of CGMs produced to date (Fig. 1). Current FDA-approved disposable sensors have a 7-to-15-day wear time and increased accuracy without need for calibration or confirmatory blood glucose testing^8^ (Table 1). CGM transmission technology has also expanded to allow CGM data to be communicated to a user’s phone or CGM receiver via bluetooth transmission (Fig. 2).

**Table 1:** Comparison of currently available CGM systems.

	Dexcom G6	Dexcom G7	Libre 2+	Libre 3	Eversense E3	Medtronic Guardian 4
Accuracy (MARD)	Pediatrics: 7.7–10.1%	Ages 7–17 yrs: 8.1–9.0% Ages 2–6 yrs: 9.3%	Pediatrics: 9.7% Adult: 9.2%	Overall: 7.9%	Overall 8.5%	Pediatrics: 7–17 yrs: 11.6% Adults: 10.6%
FDA approval	Ages 2 years and up	Ages 2 years and up	Ages 2 years and up	Ages 4 years and up	Adults only	Ages 7 years and up
Follow or Sharing Data option	Yes, Dexcom Follow	Yes, Dexcom Follow	No, when used with AID system, data only available from the AID system	Yes, LibreView	Yes, Eversense NOW	Yes, Guardian Connect
Insertion Device	Auto-applicator, one touch sensor insertion, transmitter is a separate device to be clipped to sensor	Sensor and transmitter insert together, disposable transmitter	Sensor and transmitter insert together, disposable transmitter	Sensor and transmitter insert together, disposable transmitter	Minor office procedure to insert sensor	Auto-applicator, transmitter is a separate device to be clipped to sensor
Interferences	-Hydroxyurea -Has acetaminophen blocker	-Hydroxyurea -Has acetaminophen blocker	-High dose Vit. C	-High dose Vit C	-No acetaminophen interference	-Hydroxyurea
Wear time	10 days	10 days	15 days	14 days	180 days	7 days
Warm up time	120 min	30 min	60 min	60 min	24 h	120 min
Interoperability with Currently Available AID Systems	-CamAPS FX -Control IQ -Omnipod 5 -iLet Bionic Pancreas	-Control IQ -Omnipod 5 -iLet Bionic Pancreas	-Control IQ -Omnipod 5 (Europe only at time of publication)	-CamAPS FX	None	Medtronic 780 G

Although currently CGM is not indicated for inpatient use, the improvements in CGMs have amplified clinical enthusiasm about the application of CGM in the inpatient setting. CGM approved for outpatient use was tested in typical outpatient circumstances, so accuracy of CGM within the multitude of inpatient situations has been in question. Recently there have been several new publications on patient CGM use, with several more underway.^9–11^ The current lack of data on CGM accuracy in multiple inpatient settings is partly due to the amount of time between conducting and publishing findings, as the rapid rate of new technology development is outpacing the typical timeline of randomized controlled studies needed to assess clinical outcomes and obtain FDA approval. This limitation is triggering some to think of other ways to integrate real-world evidence and person-reported outcome data into a new approach for evaluating rapidly evolving CGM technologies.^12^

## How CGM works

**Fig. 3:**
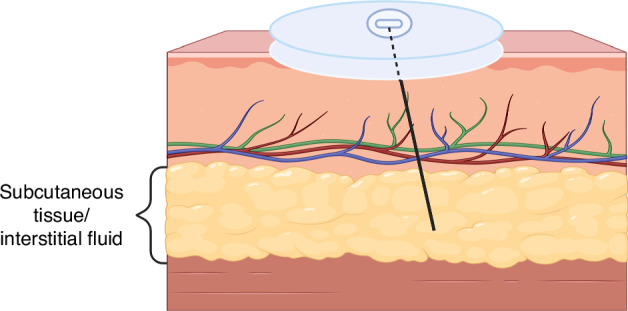
Subcutaneous CGM sideview.

**Fig. 4:**
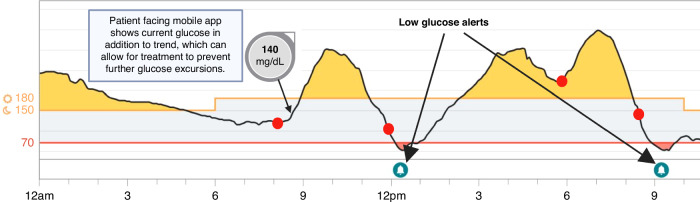
Continuous glucose monitor data display. This CGM tracing shows 4 blood glucose measurements (red dots), of which 3 are in a normal glucose range. The black line represents CGM data. However, there are large post-prandial excursions as well as 2 episodes of hypoglycemia that would be missed by the intermittent blood glucose checks. In the situations where the blood glucose checks were normal prior to hypoglycemia, if the patient saw the CGM glucose rapidly falling they may have been able to completely prevent the hypoglycemia.

An interstitial CGM system consists of three components: (1) a minimally invasive needle-type glucose biosensor electrode that measures interstitial glucose levels, (2) a transmitter to convey data wirelessly to a variety of devices and (3) a data receiver or monitor (receiver, phone, insulin pump). The thin wire-like sensor is inserted at a 45 to 90-degree angle depending on the sensor, into the subcutaneous tissue (Fig. 3) by puncturing the skin, and adhesive material keeps sensor and transmitter in place attached to the skin surface. After puncturing the skin, the insertion of the sensor causes an inflammatory reaction and a disruption of the local subcutaneous interstitial environment.^13^ Consequently, this leads to a waiting period for the sensor signal to stabilize known as “warm up time”. It varies amongst different systems, and sensor readings (glucose values) are not available for up to 30–120 min depending on the CGM system. The system monitors glucose levels continuously and is capable of predicting glucose concentrations in the future through the use of computer software algorithms. The data is displayed numerically and graphically, informing the user of glucose levels and trends (Fig. 4) every 1–5 min. The user can individualize alerts for high and low glucose levels. At the time of this writing, interstitial CGM systems use enzymatic electrochemical glucose biosensors, which have evolved over time becoming more efficient and accurate with the use of newer technologies and nanomaterials through improvements in biocompatibility and reduction of interferences.^14^

Glucose biosensing technology was discovered by Dr Leland C Clark Jr in 1965.^15^ This technology employs electrodes with redox enzymes, either glucose oxidase or dehydrogenase, the former being more selective towards glucose and able to better withstand changes in pH and temperature, hence it is the enzyme most used in commercially available CGMs. The redox reaction creates an electric current with measurable voltage that is dependent on glucose concentration. The electric signal thus formed is then transformed into a glucose value. Since the redox enzyme glucose oxidase cannot directly establish a communication with the surface of the electrode during the redox reaction, shuttling of electrons to the surface of the electrode requires a redox relay process. It is partly this electron shuttle system that has evolved and transformed glucose biosensing technology.

### Lag time and interferences

Lag time refers to CGM (interstitial glucose) readings that are delayed compared to fingerstick blood glucose readings. Glucose moves passively from the capillaries into the interstitium. Blood flow, endothelial permeability, plasma glucose levels and uptake by surrounding cells affects interstitial glucose concentrations.^16^ In steady states both serum and interstitial glucose levels correlate, with a lag time estimated at 8–10 min, but reported up to 45 min in the literature, with variability between individuals and different CGM systems.^17^ The total delay in time can be divided into physiologic and technological lag. Physiologic lag stems from the time it takes for glucose to cross the endothelial wall and reach the sensor. On the other hand, technological delay encompasses diffusion of glucose through the membranes of the sensor, redox reaction time and application of calibration algorithms to smooth and denoise the raw signal and sensor output.^18^

There are clinical implications of lag time: rapid changes in plasma glucose cause discrepancies in glucose values between blood and interstitial fluid. For example, if blood glucose is in a hypoglycemic range due to rapid drop in blood glucose, the interstitial glucose may still be in normoglycemia and the CGM would not yet alert for hypoglycemia. The incorporation of software modules that predict glucose trajectories allows CGMs to provide alerts of downtrending and uptrending glucose levels,^19,20^ which then can prevent or treat hypoglycemia or hyperglycemia before it becomes severe (Fig. 4).

Another aspect affecting accuracy of glucose values in CGM includes biofouling and biocompatibility. Insertion of sensor in the subcutaneous tissue produces a local inflammatory reaction.^13^ As a result, the foreign body response triggers adsorption of non-specific proteins and cells to the surface of biosensor, and with time this interferes with the sensor signal and function. Different anti-biofouling coatings have been introduced along with more biocompatible material to mitigate the local inflammatory reaction.^14^ In addition, inaccuracies in CGM values occur with compression on the CGM, known as “compression hypoglycemia.” This most often occurs when someone is sleeping and body weight is directly on the sensor. Often the CGM will show a very sudden, abrupt drop in glucose. The authors advise patients to check a blood glucose to confirm hypoglycemia, and if no hypoglycemia, simply taking the weight off the sensor should fix the problem with the next glucose value.

A few chemical substances may interfere with the glucose-oxidase reaction, including acetaminophen, hydroxyurea, and ascorbic acid (Vitamin C). This interference occurs because the substances can become co-oxidized and therefore transfer more electrons to the electrodes, which falsely increases glucose levels. Previous generations of Dexcom CGM had interference with acetaminophen, but Dexcom G6 and G7 include acetaminophen blockers, so it is safe to use up to 1000 mg acetaminophen every 6 h and still maintain accurate sensor readings.^21^ High vitamin C intake (more than 500 mg daily) may lead to falsely high glucose levels even with the Freestyle Libre 3; the level of inaccuracy depends on the amount of Vitamin C in the body.^22^ Hydroxyurea is noted to lead to falsely high readings with both Dexcom^21^ and Medtronic CGMs, and CGM should not be used for treatment decisions in someone taking hydroxyurea.^23^ It is also important to consider different factors that can alter interstitial glucose concentrations and hence accuracy, such as medications,^24^ pH, hydration status, hypothermia, and tissue oxygen concentration.^25^ These factors become more pertinent in hospitalized settings, where patients suffer different conditions that may impact the interstitial fluid milieu.

### Sensor accuracy

Sensor accuracy is measured by calculating the Median Absolute Relative Difference (MARD)^26^ between the sensor glucose and reference glucose value, which is the standard method used to determine accuracy for both fingerstick glucose and CGM values. A larger MARD indicates greater discrepancies between CGM and blood glucose reference values. Through the past 20 years CGM MARD has decreased with newer technology demonstrating MARD < 10%^27–31^(Fig. 1). This improvement in accuracy has led to the use of CGM without the need to confirm glucose values with a fingerstick in order to make treatment decisions, and integration into automated insulin delivery systems.

Other forms of analysis involve grids such as Clark Error Grid, Parkes Error Grid and Surveillance Error Grid, of which the former is the most commonly use in CGM studies. Grids are used to assess clinical reliability of blood glucose values with a reference glucose value divided into five clinical-decision zones: Zones A through E. Values in Zone A are within 20% of reference and therefore accurate, values in Zone B are outside of 20% range but would not result in inappropriate treatment. Zone E values would result in erroneous treatment of hyper or hypoglycemia. Conventionally, values that fall in Clark Error Grid Zone A and B are clinically accepted.^32^

## The benefits of CGM

There are many benefits of CGM. These include less fingerprick glucose checks for the patient, more glucose data which leads to more accurate insulin dose and other recommendations, ability for remote patient monitoring, integration with pumps and automated insulin delivery, less hypoglycemia, and greater time in target glucose range, all leading to improved glycemic control. When compared to capillary point-of-care (POC) testing, CGM provides data on the daily excursions of glucose as opposed to a one-time value provided by capillary POC testing. The difference in the amount of information between CGM and POC capillary testing can be likened to a movie, in comparison to photo snapshots that show only a few moments in time (Fig. 4). This wealth of glucose information has led to the many successes achieved in the outpatient setting, with improvement of glycemic control related to the incorporation of glucose trends and alerts and the ability for automated insulin delivery.

### Glycemic benefits

The first large, randomized outpatient CGM study was published in 2008^33^; it was a multicenter trial enrolling 322 adult and pediatric participants to CGM or home blood glucose monitoring. The authors demonstrated that the adults ages 25 and older had significant improvement in HbA1c, but ages 15–24 years and 8–14 years did not have improvement in HbA1c. CGM wear time of 6 days a week or more was much lower in ages 15–24 years and 8–14 years than in adults ages 25 years and older, and the differences in CGM use were a key factor associated with the age based differences in change in HbA1c. At the time, the CGM devices used in this study were all early generation; they were larger than current devices, and they required fingerstick calibrations in addition to fingerstick glucose checks for any treatment decisions, including insulin dosing or treating hypoglycemia. Burdens identified by participants included insertion pain, frequent and sometimes inaccurate alarms, and skin or body issues.^34^ This meant that for many people living with diabetes, the early generation CGMs actually increased burden, leading to low CGM wear time.

In the years since the landmark JDRF CGM^33^ study was published, substantial improvements in CGM technology have led to a rapid increase in CGM use. These improvements include factory calibration in 2015 and non-adjunctive use in 2016, as well as integration with automated insulin delivery systems starting in 2016. Data from the US based Type 1 Diabetes (T1D) Exchange registry shows that CGM use in youth increased from 4% in 2013 to 31% in 2017,^35^ and the most recent data from the type 1 diabetes exchange QI registry shows median CGM use in 2021 of 66%, and up to 81% in 2022.^36^

CGM use leads to improvement in glycemic control in nearly all age groups. Randomized studies of CGM use in adolescents and young adults show HbA1c improvements of 0.4–0.76% in the CGM groups.^37^ Data from the T1D Exchange from 2016 to 2018 showed lower HbA1c in CGM users across all ages.^7^ CGM initiated within the first year of T1D diagnosis shows long term benefit, with those who start CGM in the first year demonstrating lower HbA1c throughout 7 years of follow up compared to those who started CGM later or did not use CGM.^38^ CGM used together with automated insulin delivery (AID) leads to even larger improvements in glycemic control when compared to CGM alone or CGM with standard insulin pump therapy. This holds true across all ages, from preschoolers to older adults, with improvements in HbA1c of 0.4–0.7%.^39–42^

Improvement in glycemic control reduces the risk of short-term and long-term diabetes-related complications, highlighting this technology’s long term cost-effectiveness despite the upfront high cost. ISPAD^1^ as well as ADA^2^ and EASD^3^ include CGM use as a standard of care for glucose monitoring in type 1 diabetes. ISPAD has specific guidelines regarding lower resourced countries,^43^ with glucose monitoring recommendations for 3 levels of care^44^: Comprehensive Care includes constant CGM use when possible and if not possible, intermittent CGM use; Intermediate Care recommends CGM use once every 3–6 months to identify glucose patterns; Minimum Care does not include CGM use.

In the United States, CGM coverage has expanded since the early 2020 s, and now Medicare and nearly all state Medicaid programs cover CGM for people with T1D. Many countries with government funded universal health care insurance have also recently started to cover CGM. The United Kingdom launched universal CGM coverage for T1D in 2022, after which CGM use increased from 4% in 2017 to 95% in early 2024, with pediatric T1D patients achieving HbA1c under 7.5% increasing from 28% in 2017 to 41% in 2024.^45,46^ Similarly, after the Australian Government funded CGM technology for those with T1D under 21 years of age in April 2017, the usage rate increased from 5% to 79%, and was maintained up to 2 years afterwards.^47^ CGM was fully subsidized for children under 10 years of age, and for those 10 to <21 years if they had significant hypoglycemia. Over the 24 months following CGM subsidy, patients with CGM use >75% showed a decrease in HbA1c in addition to fewer episodes of DKA and severe hypoglycemia.

### Psychosocial benefits

Equally important to improvements in glycemic control are the improvements in quality of life for children living with T1D and their families. The use of CGM is associated with an increase in quality-adjusted life years in studies done in adult patients.^48,49^ There is a relief of burden related to not having to do frequent (painful) fingerstick checks, and mitigation of fear of hypoglycemia with predictive alarms.^50,51^ Parents have a sense of control of their child’s diabetes, which allows for more normalcy in everyday life.^50^ Using CGM with remote monitoring improves multiple measures of quality of life, reduces family stress, and improves parental sleep.^52^ Prevention of major symptomatic hypo- and hyperglycemic episodes has the potential to improve school attendance and participation in age-appropriate activities.

### Barriers of CGM use

Despite the benefits, there are barriers to wearing a CGM for many patients. These barriers include not wanting to stand out as different in social settings, dislike of technical devices on the body, and concerns that the technology is not accurate. Some patients mistrust data sharing with their healthcare team. For others, CGM utilization is limited because of poor device adhesion to skin resulting in more frequent sensor changes and running out of supplies, or local allergic reactions to adhesive, all of which can increase CGM-related costs.^53^ Alarms can be distressing, and the constant availability of glucose data may overwhelm parents and provoke anxiety related to the management of low or high glucose values.^54^

The cost of CGM is a primary barrier for many at an individual and country level. In the United states in 2024, the cost of 1-month supply of Dexcom G7 for individuals without insurance is priced at 171.28–411.20 US Dollars and For FreeStyle Libre 3 at 135.99–153.76 US Dollars.^55,56^ In countries where government sponsored health care exists, subsidy of CGM is most likely to be cost-effective.^48,49,57^ As long as the cost remains high and unsubsidized, the disparity in diabetes care will continue to widen. An alternative solution consisting of planned intermittent use of CGM for special circumstances (management of acute complications, treatment intensification, summer camps) or for select patients (hypoglycemia unawareness, pregnancy) may lessen this effect and increase global affordability.^58^

## The use of CGM in the inpatient setting

Point of care (POC) capillary blood glucose testing remains the current standard for monitoring dysglycemia in patients who are hospitalized. However, POC capillary blood glucose was originally designed and approved for home management of diabetes. The introduction and approval in the inpatient setting was controversial and met with many barriers, it was used off label in many hospital settings.^59,60^ Inpatient use of POC glucose testing was not FDA approved until 2018,^61^ which closely mirrors the current experience of inpatient CGM use; despite its wide and standard use in the ambulatory space for management of diabetes, initiating a CGM device in the hospital to monitor dysglycemia has not been approved by the FDA. Current ADA guidelines support its use in patients with T1D who are wearing a CGM device at the time of admission “as long as resources and training are available, and according to an institutional protocol, with the exception of patients with extensive skin infections, hypoperfusion, or hypovolemia or those receiving vasoactive or pressor therapy since it can potentially affect CGM accuracy”.^62^

The ever-evolving CGM technology is poised to have significant impact in the inpatient clinical setting. Most studies to date have been conducted in the adult population, focusing on comparing accuracy of CGM in different inpatient settings in both preventing hypoglycemia and treating glucose excursions in patients with diabetes,^63^ use of CGM in non-diabetes patients in the ICU to monitor hyperglycemia and maintain tight target glucose ranges,^64^ and intraoperative management of dysglycemia.^65^

On the contrary, studies in the pediatric population lag behind. This lack of data is partly explained by the general limitations of pediatric CGM utilization. CGM is not approved for children under 2 years of age, glucose readings are limited to 40–400 mg/dL, and they are not as reliable in situations of rapid blood glucose changes. Therefore its utility may be limited in pediatric patients with hyperglycemia emergencies or hypoglycemia, which are the most common scenarios requiring frequent glucose monitoring in hospitalized pediatric patients. During the COVID pandemic, the glucose monitoring requirements for the large volumes of high risk patients led to temporary FDA approval for inpatient CGM use.^66^ This rapidly accelerated the pace of inpatient CGM use, but the pediatric population was not affected in the same magnitude, and as a result there were fewer inpatient pediatric CGM studies during the pandemic.

This temporary FDA policy^66^ allowed many institutions to take advantage of CGM technology - continuous monitoring and streaming of glucose- in ICU and non-ICU settings, with the aim of minimizing healthcare worker exposure time and saving personal protective equipment.^67^ Pilot studies that emerged during the pandemic looked at accuracy of CGM devices in non-critically ill patients provided reassuring results.^67^ A randomized study comparing a CGM guided hypoglycemia management protocol to standard of care showed that a glucose telemetry system decreased hypoglycemia for insulin treated adults with type 2 diabetes.^68^ Fortman et al.^69^ looked at the utility and barriers of streaming continuous glucose readings, proposing that streaming of glucose provides valuable information and it should be regarded as the 5th vital sign. From these studies and COVID experience, we learned that implementation approaches such as training of staff, device set-up, data transmission and sharing, documentation of CGM glucose values, guideline protocols to respond to glucose data trends, feasibility of routine use and acceptability in the critical and non-critical workspace have been understudied.^70^ Nonetheless, adaptation of integration of CGM technology in the hospitals is the next vision.^71^

Frechman et al.^72^ in his 2023 review of clinical performance evaluation of CGM systems in the hospitals proposed a framework for reporting the design and results of a clinical CGM performance evaluation. The same year, the Diabetes Technology Society formulated expert recommendations for CGM implementation in the hospital based on outcome data as well as shared collective real-life experiences.^71^ Meeting sections included implementation of protocols, financial implications, development of quality metrics, data integration into EMR among others. The standardizing of glucose metrics and definitions of outcomes such as hospital time-in-range and glycemic variability index will lead to improved comparability between studies of CGM performance. Several societies have already recommended guidelines on the use of CGM in the various hospital settings, which include CGM indications and glycemic targets.^73^

### Use of CGM in the inpatient pediatric setting

The potential utility of CGM in the pediatric population includes children with diabetes, but also extends beyond diabetes settings, such as looking at glucose trends in children without diabetes experiencing hyperglycemia when treated with steroids, undergoing chemotherapy, and in pediatric patients admitted for diagnostic fasts to rule out a metabolic disorder.

There is very limited data on accuracy and implementation of CGM in hospitalized pediatric patients. Pre-pandemic pediatric studies looking at accuracy of CGMs in critically ill pediatric patients were conducted before 2015, at a time when CGM was not factory-calibrated and MARD in the outpatient setting was >10%.^74–76^ They were limited by the small sample, and as expected MARD values were high, indicating that accuracy was not good enough to use CGM values for insulin dosing or treating hypoglycemia without confirmatory capillary glucose testing. Nonetheless, they introduced CGM implementation as a tool to decrease frequency of fingerstick checks, and in this way not only eased the burden of nursing staff but improved the quality of sleep in hospitalized pediatric patients.

A retrospective chart review of hospitalized pediatric patients with T1D^10^ already wearing a CGM, who had been admitted to PICU or medical floor for either a diabetes related concern or another acute illness had concluding results similar to adult data, demonstrating a clinically acceptable accuracy of the Dexcom G6. As expected from adult studies, accuracy decreased with hypoglycemia, however in the setting of hyperglycemia accuracy was very good with a MARD of 5.6%. The accuracy was better in the ICU than on the medical floor, a possible explanation by the authors is that those patients in the ICU may have less fluctuating values of glucose due to factors such as decreased oral carbohydrate intake and activity, therefore limiting lag time between CGM and blood values.

Another recent prospective study^77^ involving 35 children hospitalized with DKA showed that CGM values were comparable to POC and serum glucose measurements, with acceptable Clark Error Grid accuracy. In addition, the authors reported that low levels of bicarbonate and impaired perfusion during DKA did not seem to impact CGM accuracy.^77^ For this study, Dexcom G6 was placed upon arrival to the PICU to the abdomen. Treatment of DKA was initiated prior to placement and use of CGM. POC and CGM data were matched up after the 2-hour warm up time and when BG had fallen <400 mg/dl. CGM was removed after closure of anion gap and bicarbonate level was >18 mEq/L. Another retrospective chart review study by Waterman et al.^9^ evaluated the accuracy of Dexcom G6 in 38 pediatric patients with T1D treated with insulin infusions in the PICU, demonstrating similar findings. The use and implementation of CGM in the setting of DKA has its own limitations beyond accuracy. For example, CGM technology cannot provide glucose readings >400 mg/dl, and warm up time delays the timing of first CGM reading.

More studies looking at CGM use in different hospital settings and pathological conditions are needed. This includes studies on accuracy, cost-effectiveness, and implementation of CGM alerts and trend predictions into hospital protocols. Guidelines are needed to manage hyperglycemia emergencies and hypoglycemia prevention on the medical floors, as well maintaining glycemic control in critically ill pediatric patients. Future studies should be conducted following similar methods and standards to measure accuracy in different hospital settings (PICU, NICU, general wards, or operating rooms) and age groups. Additionally pediatric patients may require different glycemic metric targets according to diagnosis (diabetes, chemotherapy treatment, hyperinsulinemia, steroid-induced hyperglycemia). Studies should include evaluation of acceptance by nursing staff as an adjunctive tool to guide 24-h management (Table 2).

**Table 2:** Pediatric studies of CGM use in different inpatient settings.

Study author, year	Study design	Population description	CGM	Accuracy measurement	Primary outcome
Cobry et al.^10^	Retrospective 3- year Chart Review	*N* = 83 Children with T1D during ICU and non-ICU encounters, median age 12 y	Dexcom G6	MARD POC-CGM matched pairs: Overall 11.8%. PICU: 7.9%. Medical floor: 13.5% Lab BG- CGM matched pairs: Overall 6.5%	CGM accuracy in hospitalized pediatric patients
Pott et al.^77^	Prospective, single-arm, single center	*N*: 35 Children 2–18 years with existing or new onset T1D, presenting in DKA	Dexcom G6	Clarke Error Grid POC-CGM matched pairs A + B 95.4% Serum BG-CGM matched pairs A + B 95.6%	Accuracy of CGM glucose values compared to POC and serum glucose values during standard treatment of DKA
Waterman et al. (2023)	Retrospective Chart Review	*N*: 83 Children with T1D admitted for DKA, median age 12 y, various severities of DKA	Dexcom G6	CEG during IV insulin infusion POC-CGM matched pairs A + B 98.3% MARD 15.3%	CGM accuracy during IV insulin infusion
Galderisi et al.^83^	RCT	*N*: 50 Newborns born at ≤32 weeks’ gestation or with birth weight ≤1500 g recruited within 48 hours of life	Dexcom G4	n/a	Effectiveness of CGM vs standard of care blood glucose monitoring for guiding and maintaining euglycemia in very preterm infants
Beardsell et al. (2021)	RCT – International study	*N*: 155 Newborns born at < 34 weeks’ gestation or with birth weight ≤1200 g recruited within 24 h of life	Medtronic Enlite	n/a	Effectiveness of CGM to guide the clinical management of glycemic control and use of insulin in preterm infants.

### Use of CGM in the neonatal and preterm population

A handful of studies in the last decade have looked at the role of CGM technology in the NICU, especially in the premature and very low birth weight (VLBW) population, who are prone to have greater glucose variability in the first week of life. Continuous glucose monitoring when compared to intermittent glucose monitoring has the advantage to closely assess glycemic fluctuations and hence serve as an adjunctive tool to guide treatment management to reduce hypoglycemia and/or sustained hyperglycemia^78,79^ both of which are associated with neonatal morbidities^80^ and impaired neurodevelopmental outcomes in childhood.^81^ There are no CGMs specifically designed or approved to use in neonates, but as the size decreases their use also becomes more feasible. There is great variability in measuring accuracy and in accuracy results. Studies evaluating the accuracy of CGMs are few, with most conducted with older CGMs.^82^ This remains a gap in the literature.

Galderisi et al. in 2017^83^ aimed to assess effectiveness of CGM-guided glucose administration vs standard of care blood glucose monitoring in increasing time spent in euglycemia and subsequently reducing hypoglycemia and hyperglycemia in preterm infants <32 weeks and under 1500 grams. This was a randomized controlled trial that included 50 newborns <48 h of life. The study was conducted with Dexcom G4 CGM, which was placed on the lateral thigh and worn for up to 7 days. The authors demonstrated the effectiveness of a CGM-guided algorithm for glucose infusion titration to maintain glucose control without the need for insulin.

The international, multicenter, open-label randomized controlled trial, real-time continuous glucose monitoring in preterm infants (REACT)^84^ ran from 2016 to 2019 and was the first study of its kind to evaluate the efficacy, safety and use of CGM in preterm infants. The primary aim was to evaluate the use of CGM in guiding the clinical management of glycemic control and use of insulin in preterm infants. The clinical protocol^85^ included modifications of the rate of dextrose infusion or use of insulin. CGM data was collected from a total of 155 patients <24 h of life and <34 weeks of gestational age with a birth weight of 1200 g or less. The intervention lasted until 7 days of age, but clinical outcome data was collected until 36 weeks corrected gestational age. The CGM used was Medtronic Enlite, inserted in the lateral aspect of the thigh. In this study, the use of CGM during the first week of life facilitated earlier detection of and prevention of exposure to the extremes of hypoglycemia and hyperglycemia, in addition to increasing time in the target range for glucose. The rate of necrotizing enterocolitis was also higher in the control (standard-care) group, but the study was not powered to provide statistical significance. CGM accuracy was not assessed, in turn, the protocol provided safety measures during rapid drops or increases of glucose. No skin problems were reported, and 7-day sensor wear was well tolerated (Table 2).

Implementation of continuous glucose monitoring in preterm infants is associated with a high probability of cost-effectiveness, which has the potential to improve if larger studies confirm that CGM use in preterm VLBW infants reduces the risk of pathologies thought to be associated with hyperglycemia in the first weeks of life, such as necrotizing enterocolitis, retinopathy of prematurity and possibly bronchopulmonary dysplasia.^84,86^

The NICU population has unique characteristics, differing from older pediatric and adult patients such as skin fragility and decreased subcutaneous tissue. In the studies reviewed for this writing, there were no reports about infection, skin breakdown or inflammation in sensor sites, even after the sensor was removed. Sensor wear did not last more than 7 days and they were usually placed in thighs. Additionally, CGM use in the NICU setting may provide reduced procedural pain by not only decreasing frequency of heelsticks, but insertion of the CGM cannula may be less painful than a single heelstick.^87^

## CGM as diagnostic or managing tool

CGMs as a diagnostic tool for pre-symptomatic type 1 diabetes and cystic fibrosis related diabetes has the potential to replace the oral glucose tolerance test (OGTT) which is currently the gold standard.

Type 1 diabetes is now known to be a disease that progresses in three stages, with stage 1 characterized by autoimmunity but normoglycemia, followed by stage 2 with dysglycemia, and finally stage 3 symptomatic diabetes. The use of OGTT has limitations for assessing progression to stage 3 T1D, since it is difficult to perform in young children, and parents might not agree to the required repeated tests to assess for dysglycemia. Not only can CGM be used for monitoring glycemic profiles and predicting progression to stage 3 Diabetes^88,89^ but is more acceptable to children and their families than the OGTT or blood tests. This is clinically relevant given the recent FDA approval of teplizumab, a humanized anti-CD3 monoclonal antibody, for the prevention of progression of stage 2 to stage 3 T1D.

There are limited studies exploring the utility and implementation of CGM technology in youth with Cystic Fibrosis related diabetes (CFRD). Screening for CFRD as early as 10 years of age using the OGTT is recommended.^89^ Mainguy et al.^90^ used CGMs as a tool to classify dysglycemia in youth with CF, comparing the sensitivity and specificity of different dysglycemia screening methods to OGTT. CGM data revealed that glucose excursions were prevalent early in life. Although not currently used to diagnose CFRD, CGM-detected dysglycemia among individuals with CF and normal glucose tolerance correlates with early abnormalities in insulin secretion and declines in pulmonary function.^91^ Future studies investigating the impact of CGM on glycemic control, pulmonary function, weight, or quality of life in patients with CFRD are needed.^92^

A growing interest in the use of CGM has also permeated other areas of Pediatric Endocrinology such as congenital hyperinsulinism (CHI), a disorder characterized by severe hypoglycemia due to dysregulated insulin secretion. Patients with this condition need to have up to 4–6 daily fingersticks to measure plasma glucose, but this current gold-standard approach may fail to detect episodes of hypoglycemia that could negatively impact neurodevelopmental outcomes.

When Conrad et al. in 2004^93^ assessed the use of Medtronic CGM in patients with CHI, the authors found that CGM was more useful in documenting euglycemia than hypoglycemia. A similar study by Alsaffar et al.^94^ in 2018 using Freestyle Libre found significant variability between CGM and glucose meter measurements in addition to finding a MARD of 17.9%. Another study in 2019 by Rayannavar et al.^95^ showed a similar MARD of 17.4%. In their findings, although a high false positive rate of hypoglycemia was reported, a high negative predictive value of a CGM reading for hypoglycemia was noted, proposing the utility of CGMs as a potential adjunctive tool in patients with CHI in verifying euglycemia and alerting parents when the child is not euglycemic, more importantly overnight.

One limitation for research in congenital hyperinsulinism is the lack of accuracy criteria of CGM in patients by MARD or error grids. In 2022, Worth et al. developed^96^ the Hypoglycemia Error Grid (HEG). This tool was applied in a subsequent study enrolling 10 patients over 12 weeks, using the Dexcom G6, which has a lower MARD than the CGM systems used in previous studies. The study analysis demonstrated insufficient accuracy and low rates of hypoglycemia detection. In their conclusions, the use of CGM as a standalone tool was not recommended for patients with congenital hyperinsulinism.

## The future of glucose monitoring

Interstitial continuous glucose monitoring has allowed us to learn about dynamic changes in glucose levels. This window of opportunity and biotechnological innovation brought about changes that have improved the management of diabetes in the outpatient space, and are now making their way to the inpatient setting. Next on the horizon is the expansion of indications for CGM use (younger ages, pregnancy, intraoperative, general hospital and ICU settings), as well as further development of CGM algorithms and automated insulin delivery systems. In addition, the use of other biologics could open the opportunity for non-invasive glucose monitoring which could further improve quality of life by reducing painful procedures to track glycemia. Researchers are honing in on the development of devices that detect glucose in sweat, saliva, breath, and ocular fluid as well as with the use of light. Ocular glucose sensors have been developed in the form of both contact lenses and a coil in the eyelid, and sweat sensors in the form of tattoos, patches or adhesive tapes. Non-invasive intermittent glucose monitoring is being developed with measurements of compounds in breath, use of light via Raman Spectroscopy, and even saliva in the form of a disposable test strip (biosensor) that changes color or attaches to a smartphone, which may be more cost-effective in resource-restricted areas or in certain populations.^14,15,19,97,98^

## Conclusion

The future vision of CGM technology use in the medical field is ramping up, with studies showing reliable results in multiple areas and design of interstitial CGM-mediated management innovations. The expansion of CGM for multiple settings and indications is underway, as well as ongoing development of new technologies and algorithms to continue to improve ease of use and accuracy. The use of these new and rapidly changing technologies comes with other challenges: knowledge of technology, training of staff, development of protocols to address alerts and predictions (trends). These barriers can be more accentuated in community hospitals without a dedicated endocrinology team. Implementation research will be an ongoing necessity and should be incorporated in future CGM studies.

## References

[1] Limber Ispad 2022 Con- Sensus Guidelines Chapter 7, Section. *Pediatr. Diabetes* **23**, 1243–1269 (2022). 10.1111/pedi.13417 36537530

[2] ElSayed, N. A. et al. 7. Diabetes Technology: Standards of Care in Diabetes—2023. *Diabetes Care* **46**, S111–S127 (2022). 10.2337/dc23-S007 PMC9810474 36507635

[3] Holt, R. I. et al. The Management of Type 1 Diabetes in Adults. A Consensus Report by the American Diabetes Association (Ada) and the European Association for the Study of Diabetes (Easd). *Diabetes Care* **44**, 2589–2625 (2021). 34593612 10.2337/dci21-0043

[4] Gross, T. M. et al. Performance Evaluation of the Minimed Continuous Glucose Monitoring System during Patient Home Use. *Diabetes Technol. Ther.* **2**, 49–56 (2000). 11467320 10.1089/152091500316737

[5] Hirsch, I. B. Introduction: history of glucose monitoring. *ADA Clinical Compendia* 2018;2018:1. 34251770

[6] FDA Advisory Panel Votes to Recommend Non-Adjunctive Use of Dexcom G5 Mobile CGM. *Diabetes Technol. Ther.* **18**, 512–516 (2016). 10.1089/dia.2016.07252.mr 27472488

[7] Foster, N. C. et al. State of Type 1 Diabetes Management and Outcomes from the T1d Exchange in 2016–2018. *Diabetes Technol. Therapeutics* **21**, 66–72 (2019). 10.1089/dia.2018.0384 PMC7061293 30657336

[8] American Diabetes Association. 7. Diabetes Technology: Standards of Medical Care in Diabetes-2020. *Diabetes Care* **43**, S77–s88 (2020). 10.2337/dc20-S007 31862750

[9] Waterman, L. A. et al. Accuracy of a Real-Time Continuous Glucose Monitor in Pediatric Diabetic Ketoacidosis Admissions. *Diabetes Technol. Therapeutics* **26**, 626–632 (2024). 10.1089/dia.2023.0542 PMC11535449 38441904

[10] Cobry, E. C. et al. Accuracy of a Continuous Glucose Monitor during Pediatric Type 1 Diabetes Inpatient Admissions. *Diabetes Technol. Ther.* **26**, 119–124 (2024). 38194229 10.1089/dia.2023.0375 PMC11535460

[11] Cobry, E. CGM during Surgery in the Pediatric Population. In *Oral Presentation ADA, Orlando Fl* (ADA, 2024).

[12] Rickson, M., Wright, E. E. Jr., Bindal, A. & Ghonim, L. Advancements in Diabetes Technology Are Outpacing the Evidence. *Diabetes Technol. Ther.* **25**, S35–s41 (2023). 37306447 10.1089/dia.2023.0145

[13] Joseph, J. I. et al. Glucose Sensing in the Subcutaneous Tissue: Attempting to Correlate the Immune Response with Continuous Glucose Monitoring Accuracy. *Diabetes Technol. Ther.* **20**, 321–324 (2018). 29792751 10.1089/dia.2018.0106 PMC6110119

[14] Teymourian, H., Barfidokht, A. & Wang, J. Electrochemical Glucose Sensors in Diabetes Management: An Updated Review (2010-2020). *Chem. Soc. Rev.* **49**, 7671–7709 (2020). 33020790 10.1039/d0cs00304b

[15] Lee, I., Probst, D., Klonoff, D. & Sode, K. Continuous Glucose Monitoring Systems - Current Status and Future Perspectives of the Flagship Technologies in Biosensor Research. *Biosens. Bioelectron.* **181**, 113054 (2021). 33775474 10.1016/j.bios.2021.113054

[16] Koschinsky, T. & Heinemann, L. Sensors for Glucose Monitoring: Technical and Clinical Aspects. *Diabetes Metab. Res Rev.* **17**, 113–123 (2001). 11307176 10.1002/dmrr.188

[17] Cengiz, E. & Tamborlane, W. V. A Tale of Two Compartments: Interstitial Versus Blood Glucose Monitoring. *Diabetes Technol. Ther.* **11**, S11–S16 (2009). 19469670 10.1089/dia.2009.0002 PMC2903977

[18] Schmelzeisen-Redeker, G. et al. Time Delay of Cgm Sensors: Relevance, Causes, and Countermeasures. *J. Diabetes Sci. Technol.* **9**, 1006–1015 (2015). 26243773 10.1177/1932296815590154 PMC4667340

[19] Zou, Y. et al. Minimally Invasive Electrochemical Continuous Glucose Monitoring Sensors: Recent Progress and Perspective. *Biosens. Bioelectron.* **225**, 115103 (2023). 36724658 10.1016/j.bios.2023.115103

[20] Facchinetti, A. et al. Real-Time Improvement of Continuous Glucose Monitoring Accuracy: The Smart Sensor Concept. *Diabetes Care* **36**, 793–800 (2013). 23172973 10.2337/dc12-0736 PMC3609535

[21] *Dexcom Interference Manual*, https://www.dexcom.com/interference (2023).

[22] *Abbott Libre Safety Information*, https://www.freestyle.abbott/us-en/safety-information.html. Accessed July 1 2024.

[23] *Medtronic User Manual*, https://www.medtronic.com/content/dam/medtronic-wide/public/canada/products/diabetes/780g-gs3-system-user-guide.pdf. Accessed July 1 2024.

[24] Heinemann, L. Interferences with Cgm Systems: Practical Relevance? *J. Diabetes Sci. Technol.* **16**, 271–274 (2022). 34911382 10.1177/19322968211065065 PMC8861798

[25] Herzig, D. et al. Performance of the Dexcom G6 Continuous Glucose Monitoring System during Cardiac Surgery Using Hypothermic Extracorporeal Circulation. *Diabetes Care* **46**, 864–867 (2023). 36809308 10.2337/dc22-2260

[26] Bailey, T. S. & Alva, S. Landscape of Continuous Glucose Monitoring (Cgm) and Integrated Cgm: Accuracy Considerations. *Diabetes Technol. Ther.* **23**, S5–s11 (2021). 34546084 10.1089/dia.2021.0236

[27] Bailey, T., Bode, B. W., Christiansen, M. P., Klaff, L. J. & Alva, S. The Performance and Usability of a Factory-Calibrated Flash Glucose Monitoring System. *Diabetes Technol. Ther.* **17**, 787–794 (2015). 26171659 10.1089/dia.2014.0378 PMC4649725

[28] Garg, S. K. et al. Accuracy and Safety of Dexcom G7 Continuous Glucose Monitoring in Adults with Diabetes. *Diabetes Technol. Ther.* **24**, 373–380 (2022). 35157505 10.1089/dia.2022.0011 PMC9208857

[29] Keenan, D. B., Cartaya, R. & Mastrototaro, J. J. Accuracy of a New Real-Time Continuous Glucose Monitoring Algorithm. *J. Diabetes Sci. Technol.* **4**, 111–118 (2010). 20167174 10.1177/193229681000400114 PMC2825631

[30] Christiansen, M. P. et al. A Prospective Multicenter Evaluation of the Accuracy of a Novel Implanted Continuous Glucose Sensor: Precise Ii. *Diabetes Technol. Ther.* **20**, 197–206 (2018). 29381090 10.1089/dia.2017.0142 PMC5867508

[31] Keenan, D. B. et al. Accuracy of the Enlite 6-Day Glucose Sensor with Guardian and Veo Calibration Algorithms. *Diabetes Technol. Ther.* **14**, 225–231 (2012). 22145851 10.1089/dia.2011.0199

[32] Clarke, W. L., Cox, D., Gonder-Frederick, L. A., Carter, W. & Pohl, S. L. Evaluating Clinical Accuracy of Systems for Self-Monitoring of Blood Glucose. *Diabetes Care* **10**, 622–628 (1987). 3677983 10.2337/diacare.10.5.622

[33] Tamborlane, W. V. et al. Continuous Glucose Monitoring and Intensive Treatment of Type 1 Diabetes. *N. Engl. J. Med.* **359**, 1464–1476 (2008). 18779236 10.1056/NEJMoa0805017

[34] Tansey, M. et al. Satisfaction with Continuous Glucose Monitoring in Adults and Youths with Type 1 Diabetes. *Diabet. Med.* **28**, 1118–1122 (2011). 21692844 10.1111/j.1464-5491.2011.03368.x

[35] Miller, K. M. et al. Longitudinal Changes in Continuous Glucose Monitoring Use among Individuals with Type 1 Diabetes: International Comparison in the German and Austrian Dpv and U.S. T1d Exchange Registries. *Diabetes Care* **43**, e1–e2 (2020). 31672703 10.2337/dc19-1214 PMC7881298

[36] Prahalad, P. et al. Benchmarking Diabetes Technology Use among 21 U.S. Pediatric Diabetes Centers. *Clin. Diabetes* **42**, 27–33 (2024). 38230344 10.2337/cd23-0052 PMC10788667

[37] Laffel, L. M. et al. Effect of Continuous Glucose Monitoring on Glycemic Control in Adolescents and Young Adults with Type 1 Diabetes: A Randomized Clinical Trial. *JAMA* **323**, 2388–2396 (2020). 32543683 10.1001/jama.2020.6940 PMC7298603

[38] Champakanath, A., Akturk, H. K., Alonso, G. T., Snell-Bergeon, J. K. & Shah, V. N. Continuous Glucose Monitoring Initiation within First Year of Type 1 Diabetes Diagnosis Is Associated with Improved Glycemic Outcomes: 7-Year Follow-up Study. *Diabetes Care* **45**, 750–753 (2022). 35018417 10.2337/dc21-2004

[39] Brown, S. A. et al. Six-Month Randomized, Multicenter Trial of Closed-Loop Control in Type 1 Diabetes. *N. Engl. J. Med.* **381**, 1707–1717 (2019). 31618560 10.1056/NEJMoa1907863 PMC7076915

[40] Brown, S. A. et al. Multicenter Trial of a Tubeless, on-Body Automated Insulin Delivery System with Customizable Glycemic Targets in Pediatric and Adult Participants with Type 1 Diabetes. *Diabetes Care* **44**, 1630–1640 (2021). 34099518 10.2337/dc21-0172 PMC8323171

[41] Carlson, A. L. et al. Safety and Glycemic Outcomes during the Minimed™ Advanced Hybrid Closed-Loop System Pivotal Trial in Adolescents and Adults with Type 1 Diabetes. *Diabetes Technol. Ther.* **24**, 178–189 (2022). 34694909 10.1089/dia.2021.0319 PMC8971997

[42] Berget, C. et al. Six Months of Hybrid Closed Loop in the Real-World: An Evaluation of Children and Young Adults Using the 670g System. *Pediatr. Diabetes* **21**, 310–318 (2020). 31837064 10.1111/pedi.12962 PMC7204168

[43] Virmani, A. et al. ISPAD Clinical Practice Consensus Guidelines 2022: Management of the Child, Adolescent, and Young Adult with Diabetes in Limited Resource Settings. *Pediatric Diabetes* **23**, 1529–1551 (2022). 10.1111/pedi.13456 36537524

[44] Ogle, G. D., von Oettingen, J. E., Middlehurst, A. C., Hanas, R. & Orchard, T. J. Levels of Type 1 Diabetes Care in Children and Adolescents for Countries at Varying Resource Levels. *Pediatr. Diabetes* **20**, 93–98 (2019). 30471084 10.1111/pedi.12801

[45] Kar, P. *Oral presentation at Advanced Technologies and Treatments in Diabetes, Florence Italy* (2024).

[46] NHS Database Website, https://digital.nhs.uk/data-and-information/publications/statistical/national-diabetes-audit/e3-national-diabetes-audit-nda-2023-24-quarterly-report-for-england-integrated-care-board-icb-primary-care-network-pcn-and-gp-practice.

[47] Johnson, S. R. et al. Universal Subsidized Continuous Glucose Monitoring Funding for Young People with Type 1 Diabetes: Uptake and Outcomes over 2 Years, a Population-Based Study. *Diabetes Care* **45**, 391–397 (2022). 34872983 10.2337/dc21-1666 PMC8914416

[48] Roze, S. et al. Long-Term Cost-Effectiveness the Dexcom G6 Real-Time Continuous Glucose Monitoring System Compared with Self-Monitoring of Blood Glucose in People with Type 1 Diabetes in France. *Diabetes Ther.* **12**, 235–246 (2021). 33165838 10.1007/s13300-020-00959-y PMC7651823

[49] Alshannaq, H. et al. Cost-Utility of Real-Time Continuous Glucose Monitoring Versus Self-Monitoring of Blood Glucose and Intermittently Scanned Continuous Glucose Monitoring in People with Type 1 Diabetes Receiving Multiple Daily Insulin Injections in Denmark. *Diabetes Obes. Metab.* **25**, 2704–2713 (2023). 37334522 10.1111/dom.15158

[50] Haslund-Thomsen, H., Hasselbalch, L. A. & Laugesen, B. Parental Experiences of Continuous Glucose Monitoring in Danish Children with Type 1 Diabetes Mellitus. *J. Pediatr. Nurs.* **53**, e149–e155 (2020). 32245681 10.1016/j.pedn.2020.03.010

[51] Commissariat, P. V. et al. Twelve-Month Psychosocial Outcomes of Continuous Glucose Monitoring with Behavioural Support in Parents of Young Children with Type 1 Diabetes. *Diabet. Med.* **40**, e15120 (2023). 37083018 10.1111/dme.15120 PMC10524740

[52] Burckhardt, M. A. et al. The Use of Continuous Glucose Monitoring with Remote Monitoring Improves Psychosocial Measures in Parents of Children with Type 1 Diabetes: A Randomized Crossover Trial. *Diabetes Care* **41**, 2641–2643 (2018). 30377184 10.2337/dc18-0938

[53] Berg, A. K. et al. Cost of Treating Skin Problems in Patients with Diabetes Who Use Insulin Pumps and/or Glucose Sensors. *Diabetes Technol. Therapeutics* **22**, 658–665 (2019). 10.1089/dia.2019.0368 31800294

[54] Borges, U. & Kubiak, T. Continuous Glucose Monitoring in Type 1 Diabetes: Human Factors and Usage. *J. Diabetes Sci. Technol.* **10**, 633–639 (2016). 26961974 10.1177/1932296816634736 PMC5038544

[55] Cost of Dexcom in USA, https://www.goodrx.com/dexcom-g7. Accessed July 1 2024.

[56] Freestyle Libre 3 Cost in USA, https://www.goodrx.com/freestyle-libre-3. Accessed July 1 2024.

[57] Bahia, L. et al. Cost-Effectiveness of Continuous Glucose Monitoring with Freestyle Libre(®) in Brazilian Insulin-Treated Patients with Types 1 and 2 Diabetes Mellitus. *Diabetol. Metab. Syndr.* **15**, 242 (2023). 38001509 10.1186/s13098-023-01208-5 PMC10675900

[58] Ziegler, R. et al. Intermittent Use of Continuous Glucose Monitoring: Expanding the Clinical Value of Cgm. *J. Diabetes Sci. Technol.* **15**, 684–694 (2020). 32064909 10.1177/1932296820905577 PMC8120049

[59] FDA. *Clinical Accuracy Requirements for Point of Care Blood Glucose Meters; Public Meeting 2010* (FDA, 2010). 10.1177/193229681000400234 PMC2864188 20307413

[60] Rajendran, R. & Rayman, G. Point-of-Care Blood Glucose Testing for Diabetes Care in Hospitalized Patients: An Evidence-Based Review. *J. Diabetes Sci. Technol.* **8**, 1081–1090 (2014). 25355711 10.1177/1932296814538940 PMC4455482

[61] FDA. *Statstrip Glucose Hospital Meter System Approved for Use in Critically Ill Patients* (FDA, 2018).

[62] Committee, A. D. A. P. P. 16. Diabetes Care in the Hospital: Standards of Care in Diabetes—2024. *Diabetes Care* **47**, S295–S306 (2023). 10.2337/dc24-S016 PMC10725815 38078585

[63] Galindo, R. J. et al. Comparison of the Freestyle Libre Pro Flash Continuous Glucose Monitoring (Cgm) System and Point-of-Care Capillary Glucose Testing in Hospitalized Patients with Type 2 Diabetes Treated with Basal-Bolus Insulin Regimen. *Diabetes Care* **43**, 2730–2735 (2020). 32641372 10.2337/dc19-2073 PMC7809713

[64] Furushima, N., Egi, M., Obata, N., Sato, H. & Mizobuchi, S. Mean Amplitude of Glycemic Excursions in Septic Patients and Its Association with Outcomes: A Prospective Observational Study Using Continuous Glucose Monitoring. *J. Crit. Care* **63**, 218–222 (2021). 32958351 10.1016/j.jcrc.2020.08.021

[65] Price, C. E. et al. Feasibility of Intraoperative Continuous Glucose Monitoring: An Observational Study in General Surgery Patients. *J. Clin. Anesth.* **87**, 111090 (2023). 36913777 10.1016/j.jclinane.2023.111090

[66] American Diabetes Association: Fda Expands Remote Patient Monitoring in Hospitals for People with Diabetes during Covid-19; Manufacturers Donate Cgm Supplies. April 20, 2020.

[67] Ehrhardt, N. & Hirsch, I. B. The Impact of Covid-19 on Cgm Use in the Hospital. *Diabetes Care* **43**, 2628–2630 (2020). 32978180 10.2337/dci20-0046

[68] Singh, L. G. et al. Reducing Inpatient Hypoglycemia in the General Wards Using Real-Time Continuous Glucose Monitoring: The Glucose Telemetry System, a Randomized Clinical Trial. *Diabetes Care* **43**, 2736–2743 (2020). 32759361 10.2337/dc20-0840 PMC7576426

[69] Fortmann, A. L. et al. Glucose as the Fifth Vital Sign: A Randomized Controlled Trial of Continuous Glucose Monitoring in a Non-Icu Hospital Setting. *Diabetes Care* **43**, 2873–2877 (2020). 32855160 10.2337/dc20-1016 PMC7576427

[70] Faulds, E. R., Dungan, K. M. & McNett, M. Implementation of Continuous Glucose Monitoring in Critical Care: A Scoping Review. *Curr. Diab Rep.* **23**, 69–87 (2023). 37052790 10.1007/s11892-023-01503-5 PMC10098233

[71] Tian, T. et al. Use of Continuous Glucose Monitors in the Hospital: The Diabetes Technology Society Hospital Meeting Report 2023. *J. Diabetes Sci. Technol.* **17**, 1392–1418 (2023). 37559371 10.1177/19322968231186575 PMC10563530

[72] Freckmann, G. et al. Clinical Performance Evaluation of Continuous Glucose Monitoring Systems: A Scoping Review and Recommendations for Reporting. *J. Diabetes Sci. Technol.* **17**, 1506–1526 (2023). 37599389 10.1177/19322968231190941 PMC10658695

[73] Zelada, H., Perez-Guzman, M. C., Chernavvsky, D. R. & Galindo, R. J. Continuous Glucose Monitoring for Inpatient Diabetes Management: An Update on Current Evidence and Practice. *Endocr. Connect.* **12**, e230180 (2023). 10.1530/EC-23-0180 PMC10563639 37578799

[74] Gangu, S., Tinsley, C. & Abd-Allah, S. 909: A Study Comparing the Accuracy of Cgm Device to Fsbg Levels among Picu Patients in Dka. *Crit. Care Med.* **43**, 229 (2015).

[75] Branco, R. G., Chavan, A. & Tasker, R. C. Pilot Evaluation of Continuous Subcutaneous Glucose Monitoring in Children with Multiple Organ Dysfunction Syndrome. *Pediatr. Crit. Care Med.* **11**, 415–419 (2010). 19924024 10.1097/PCC.0b013e3181c59144

[76] Piper, H. G. et al. Real-Time Continuous Glucose Monitoring in Pediatric Patients during and after Cardiac Surgery. *Pediatrics* **118**, 1176–1184 (2006). 16951013 10.1542/peds.2006-0347

[77] Pott, T., Jimenez-Vega, J., Parker, J. & Fitzgerald, R. Continuous Glucose Monitoring in Pediatric Diabetic Ketoacidosis. *J. Diabetes Sci. Technol.*, **18**, 899–903 (2024). 10.1177/19322968221140430 PMC11307226 36416103

[78] Beardsall, K. et al. Validation of the Continuous Glucose Monitoring Sensor in Preterm Infants. *Arch. Dis. Child Fetal Neonatal Ed.* **98**, F136–F140 (2013). 22791467 10.1136/archdischild-2012-301661

[79] Fernández-Martínez, M. D. M. et al. Monitoring the Incidence, Duration and Distribution of Hyperglycaemia in Very-Low-Birth-Weight Newborns and Identifying Associated Factors. *J. Perinat. Med.* **48**, 631–637 (2020). 32432567 10.1515/jpm-2020-0074

[80] Zamir, I. et al. Hyperglycemia in Extremely Preterm Infants-Insulin Treatment, Mortality and Nutrient Intakes. *J. Pediatr.* **200**, 104–110.e101 (2018). 29731360 10.1016/j.jpeds.2018.03.049

[81] McKinlay, C. J. D. et al. Association of Neonatal Glycemia with Neurodevelopmental Outcomes at 4.5 Years. *JAMA Pediatr.* **171**, 972–983 (2017). 28783802 10.1001/jamapediatrics.2017.1579 PMC5710616

[82] Shah, R., McKinlay, C. J. D. & Harding, J. E. Neonatal Hypoglycemia: Continuous Glucose Monitoring. *Curr. Opin. Pediatr.* **30**, 204–208 (2018). 29346140 10.1097/MOP.0000000000000592 PMC5882205

[83] Galderisi, A. et al. Continuous Glucose Monitoring in Very Preterm Infants: A Randomized Controlled Trial. *Pediatrics* **140**, e20171162 (2017). 10.1542/peds.2017-1162 28916591

[84] Beardsall, K. et al. Real-Time Continuous Glucose Monitoring in Preterm Infants (React): An International, Open-Label, Randomised Controlled Trial. *Lancet Child Adolesc. Health* **5**, 265–273 (2021). 33577770 10.1016/S2352-4642(20)30367-9 PMC7970623

[85] Beardsall, K. et al. *Continuous Glucose Monitoring in Extremely Preterm Infants in Intensive Care: The React Rct and Pilot Study of ‘Closed-Loop’ Technology* (NIHR Journals Library Copyright © Queen’s Printer and Controller of HMSO, 2021). 34723449

[86] Petrou, S., Kim, S., Bond, S., Allison, A. & Beardsall, K. Cost-Effectiveness of Real Time Continuous Glucose Monitoring to Target Glucose Control in Preterm Infants. *Semin Perinatol.* **45**, 151392 (2021). 33549333 10.1016/j.semperi.2021.151392

[87] Galderisi, A. et al. Procedural Pain during Insertion of a Continuous Glucose Monitoring Device in Preterm Infants. *J. Pediatr.* **200**, 261–264.e261 (2018). 29861315 10.1016/j.jpeds.2018.03.040

[88] Joshi, K. et al. Continuous Glucose Monitoring Has an Increasing Role in Pre-Symptomatic Type 1 Diabetes: Advantages, Limitations, and Comparisons with Laboratory-Based Testing. *Clin. Chem. Lab. Med.* **62**, 41–49 (2024). 37349976 10.1515/cclm-2023-0234

[89] Steck, A. K. et al. Continuous Glucose Monitoring Predicts Progression to Diabetes in Autoantibody Positive Children. *J. Clin. Endocrinol. Metab.* **104**, 3337–3344 (2019). 30844073 10.1210/jc.2018-02196 PMC6589073

[90] Mainguy, C. et al. Sensitivity and Specificity of Different Methods for Cystic Fibrosis-Related Diabetes Screening: Is the Oral Glucose Tolerance Test Still the Standard? *J. Pediatr. Endocrinol. Metab.* **30**, 27–35 (2017). 27977404 10.1515/jpem-2016-0184

[91] Chan, C. L. et al. Continuous Glucose Monitoring Abnormalities in Cystic Fibrosis Youth Correlate with Pulmonary Function Decline. *J. Cyst. Fibros.* **17**, 783–790 (2018). 29580828 10.1016/j.jcf.2018.03.008 PMC6151303

[92] Marks, B. E., Williams, K. M., Sherwood, J. S. & Putman, M. S. Practical Aspects of Diabetes Technology Use: Continuous Glucose Monitors, Insulin Pumps, and Automated Insulin Delivery Systems. *J. Clin. Transl. Endocrinol.* **27**, 100282 (2022). 34917483 10.1016/j.jcte.2021.100282 PMC8666668

[93] Conrad, S. C., Mastrototaro, J. J. & Gitelman, S. E. The Use of a Continuous Glucose Monitoring System in Hypoglycemic Disorders. *J. Pediatr. Endocrinol. Metab.* **17**, 281–288 (2004). 15112904 10.1515/jpem.2004.17.3.281

[94] Alsaffar, H., Turner, L., Yung, Z., Didi, M. & Senniappan, S. Continuous Flash Glucose Monitoring in Children with Congenital Hyperinsulinism; First Report on Accuracy and Patient Experience. *Int. J. Pediatr. Endocrinol.* **2018**, 3 (2018). 29599801 10.1186/s13633-018-0057-2 PMC5870486

[95] Rayannavar, A., Elci, O. U., Mitteer, L. & De León, D. D. Continuous Glucose Monitoring Systems: Are They Useful for Evaluating Glycemic Control in Children with Hyperinsulinism? *Horm. Res. Paediatr.* **92**, 319–327 (2019). 32208390 10.1159/000506230 PMC7192768

[96] Worth, C. et al. The Hypoglycaemia Error Grid: A Uk-Wide Consensus on Cgm Accuracy Assessment in Hyperinsulinism. *Front. Endocrinol.* **13**, 1016072 (2022). 10.3389/fendo.2022.1016072 PMC9666389 36407313

[97] Zhang, Y., Sun, J., Liu, L. & Qiao, H. A Review of Biosensor Technology and Algorithms for Glucose Monitoring. *J. Diabetes Complic.* **35**, 107929 (2021). 10.1016/j.jdiacomp.2021.107929 33902999

[98] Reddy, V. S. et al. Recent Advancement in Biofluid-Based Glucose Sensors Using Invasive, Minimally Invasive, and Non-Invasive Technologies: A Review. *Nanomaterials* **12**, 1082 (2022). 35407200 10.3390/nano12071082 PMC9000490

